# Sphingolipid Metabolism Is Dysregulated at Transcriptomic and Metabolic Levels in the Spinal Cord of an Animal Model of Amyotrophic Lateral Sclerosis

**DOI:** 10.3389/fnmol.2017.00433

**Published:** 2018-01-04

**Authors:** Alexandre Henriques, Vincent Croixmarie, Alexandra Bouscary, Althéa Mosbach, Céline Keime, Claire Boursier-Neyret, Bernard Walter, Michael Spedding, Jean-Philippe Loeffler

**Affiliations:** ^1^Université de Strasbourg, UMR_S 1118, Fédération de Médecine Translationnelle, Strasbourg, France; ^2^INSERM, U1118, Mécanismes Centraux et Périphériques de la Neurodégénérescence, Strasbourg, France; ^3^Spedding Research Solutions SAS, Le Vesinet, France; ^4^Institut de Recherche International Servier, Suresnes, France; ^5^Institut de Génétique et de Biologie Moléculaire et Cellulaire, INSERM, U964, CNRS, UMR7104, Université de Strasbourg, Illkirch, France; ^6^Technologie Servier, Orleans, France

**Keywords:** amyotrophic lateral sclerosis, RNA-sequencing, transcriptomics, metabolomics, SOD1 mice, sphingolipids, therapy, lipids

## Abstract

Lipid metabolism is drastically dysregulated in amyotrophic lateral sclerosis and impacts prognosis of patients. Animal models recapitulate alterations in the energy metabolism, including hypermetabolism and severe loss of adipose tissue. To gain insight into the molecular mechanisms underlying disease progression in amyotrophic lateral sclerosis, we have performed RNA-sequencing and lipidomic profiling in spinal cord of symptomatic SOD1^G86R^ mice. Spinal transcriptome of SOD1^G86R^ mice was characterized by differential expression of genes related to immune system, extracellular exosome, and lysosome. Hypothesis-driven identification of metabolites showed that lipids, including sphingomyelin(d18:0/26:1), ceramide(d18:1/22:0), and phosphatidylcholine(o-22:1/20:4) showed profound altered levels. A correlation between disease severity and gene expression or metabolite levels was found for sphingosine, ceramide(d18:1/26:0), *Sgpp2, Sphk1*, and *Ugt8a*. Joint-analysis revealed a significant enrichment of glycosphingolipid metabolism in SOD1^G86R^ mice, due to the down-regulation of ceramide, glucosylceramide, and lactosylceramide and the overexpression of genes involved in their recycling in the lysosome. A drug-gene interaction database was interrogated to identify potential drugs able to modulate the dysregulated genes from the signaling pathway. Our results suggest that complex lipids are pivotally changed during the first phase of motor symptoms in an animal model of amyotrophic lateral sclerosis.

## Introduction

Amyotrophic lateral sclerosis (ALS) is a non-cell autonomous disease characterized by a severe muscle denervation and degeneration of upper and lower motor neurons. ALS has prevalence close to 7 in 100,000. Death usually occurs 1–5 years after diagnosis, but survival can exceed 10 years for 10% of patients. Etiology remains unclear for the majority of patients even though genetic mutations are known to trigger or to increase the risk of ALS. The main ALS-linked genetic mutations concern *SOD1, TARBP, FUS* genes. Expansion repeats in *C9ORF72* is also an important risk factor for ALS (Taylor et al., 2016). Altered RNA metabolism, oxidative stress, impaired axonal transport, protein misfolding, and inflammation participate to disease severity. Mutation on the *SOD1* gene was the first identified genetic cause for ALS (Rosen et al., 1993). Transgenic mice harboring mutations in the *Sod1* gene are used as models of ALS, such as the SOD1^G93A^ and the SOD1^G86R^ models. They recapitulate the main symptoms of ALS, including motor neuron degeneration, muscle denervation associated with severe paralysis and death (Ripps et al., 1995).

Multiple stages of energy and lipid metabolism dysregulations exist in ALS. Incidences of hypermetabolism and glucose insensitivity are higher in ALS and severe loss of body mass negatively affects patient's prognosis (Schmitt et al., 2014). High adiposity (Paganoni et al., 2011), dyslipidaemia (Dupuis et al., 2008; Mariosa et al., 2017), and circulating metabolic markers (Henriques et al., 2015a) are associated with survival. High caloric diets stabilize weight loss and extend life expectancy of patients under gastrostomy (Wills et al., 2014; Dorst et al., 2015). Hypermetabolism and preferential use of lipids as nutrients have been reported in SOD1 mice (Dupuis et al., 2004; Palamiuc et al., 2015). The causes of the metabolic dysfunctions in ALS remain unknown and could result from central pathologies combined with peripheral alterations. Early metabolic shift from glycolysis to beta-oxidation has been evidenced in skeletal muscles in SOD1^G86R^ mice (Palamiuc et al., 2015). SOD1^G93A^ present with glucose intolerance and impaired muscle glucose metabolism, which could be reversed by physical exercise (Desseille et al., 2017). Fibroblast from sporadic or familial ALS patients exhibits a metabolic shift from oxidative phosphorylation to glycolysis (Raman et al., 2015). Hypothalamus is a cerebral structure key for the integration of central and peripheral signals related to energy balance. Neuronal loss and altered melanocortin pathway have been described in the hypothalamus of ALS patients and can account for weight unbalance and defective energy metabolism (Vercruysse et al., 2016).

The nervous system is remarkably enriched in lipids compared to other tissues. They range from simple fatty acids to glycosphingolipids. Lipids directly contribute to cell signaling, neuronal energy balance during stress, and membrane stability/fluidity. Different research groups have reported dysregulations of lipids in the central nervous system of neurodegenerative diseases. Elevated diacylglycerols have been reported in the frontal cortex of patients diagnosed with Alzheimer's disease (Wood et al., 2016) and levels of sphingolipids, phospholipids, and neutral lipids were reported as significantly dysregulated in the central nervous system (CNS) of patients diagnosed with Parkinson's disease (Cheng et al., 2011). Genetic mutations causing disrupted lipid metabolism have been linked to neurological and neuromuscular phenotypes in human diseases, like hereditary spastic paraplegia (Dodge, 2017).

In ALS, accumulation of lipid metabolites (i.e., ceramides and cholesterol esters), occurred in the post-mortem spinal cord samples of ALS patients (Cutler et al., 2002). Accumulation of ceramide species in ALS post-mortem spinal samples was confirmed, by targeted metabolomics, in a second study (Dodge et al., 2015). Using this approach with spinal tissues from SOD1^G93A^ mice, one ceramide metabolite was upregulated at disease endpoint in this animal model of ALS. Nevertheless, at earlier disease stage, ceramides, and other sphingolipids showed marked dysregulations. We have recently reported that almost all lipid classes (e.g., triglycerides, phospholipids, sphingolipids) were strongly dysregulated at both presymptomatic and late disease stages, with metabolites showing either with an upregulation or downregulation in the spinal cord of SOD1^G86R^ mice (Henriques et al., 2015b).

Gene expression analysis by RNA-sequencing gives information on the expression levels of genes in specific cells or tissues. In ALS, several molecular pathways have been already detected as altered at transcriptional levels in the CNS of ALS patients and animal models (Henriques and Gonzalez De Aguilar, 2011). A recent study has reported that targeted metabolomics (limited to 188 metabolites) combined to targeted gene expression analysis (limited to 84 genes) was able to provide information on the regulation of branched-chain amino acid metabolism in SOD1^G93A^ mice (Patin et al., 2016). We present for the first time an integrated analysis of RNA-sequencing and lipidomic data from the spinal cord of symptomatic SOD1^G86R^ mice. These results allow an unbiased vision of the metabolic changes in the course of ALS, and pinpoint specific pathways which are pathologically important.

## Materials and methods

### Animals

Experiments were performed by authorized investigator (A67-402 to A.H.), after approval of experimental procedure by the ethic committee of the University of Strasbourg and by the ministry of higher education and research (APAFIS#2255; AL/25/32/02/13). They followed current European Union regulations (Directive 2010/63/EU). FVB/N female mice, overexpressing the SOD1^G86R^ protein (Ripps et al., 1995), were generated by breeding male SOD1^G86R^ mice with non-transgenic FVB/N female mice in our animal facility. Genotypage was performed as previously described (Ripps et al., 1995). Mice were maintained in our animal facility at 23°C with a 12 h light/dark cycle. Mice had access to water and to regular A04 rodent chow *ad libitum*. Body mass and muscle strength were analyzed on a daily basis to access disease onset. For muscle strength measurements (mean of three tests, grip test, Bioseb, Chaville, France), a mouse was placed on grid where they spontaneously gripped it with their four paws. They were then gently pulled back by the experimenter until they released the grid (mean of three tests, grip test, Bioseb, Chaville, France). A strength-meter recorded the peak grip strength of the mouse. Age and litter matched non-transgenic female mice served as control. At 95 days of age, mice were sacrificed by intraperitoneal injection with ketamine chlorohydrate (100 mg/kg) and xylazine (5 mg/kg) and intracardially perfused with PBS at 4°C. Lumbar spinal cords were quickly dissected, fresh frozen, and kept at −80°C until further analysis.

### RNA-sequencing

RNA-sequencing was performed as previously described (Henriques et al., 2017). Briefly, total RNA was extracted from frozen samples of spinal cord from 95 days old mice (*n* = 5/group). Libraries of template molecules suitable for high throughput DNA sequencing were created and reads were mapped onto mm10 assembly of mouse genome using Tophat v2.0.14 (Kim et al., 2013). Quantification of gene expression was performed using HTSeq v0.6.1 (Anders and Huber, 2010) and Ensembl release 81 database. Supervised statistical analysis for differential gene expression has been performed using R (3.3.2) and the DESeq2 Bioconductor (v3.2) library. Multiple testing was adjusted by Benjamini and Hochberg FDR correction (Benjamini and Hochberg, 1995).

After normalization and rlog transformation, hierarchical clustering (single method), and PCA (principal component analysis) was performed with the indicated subset of genes, accordingly to KEGG pathways, using JMP 11.0.0. Validation of gene expression was assessed by qPCR with a CFX96 using SYBR green Supermix reagent (BioRad), with the samples used for RNA-sequencing. Relative quantification of each gene was determined using the Biorad software and normalized to reference genes (Pol2, TBP, and 18S). Primers sequences are provided in Supplementary Table 1. Comparison between groups was studied with student's *t*-test and *p*-value < 0.05 were considered significant (PRISM 6.0b, GraphPad, San Diego, CA). Data are expressed as the mean ± SEM. RNA-sequencing data are available at the Gene Expression Omnibus (GEO) database repository, under the accession number GSE106364.

### Metabolomic analysis

Lipid extraction and UPLC/TOF-MS (ultra performance liquid chromatography/time-of-flight mass spectrometry) was performed as previously described (Henriques et al., 2015b). Briefly, spinal cord samples from 95 days old mice were homogenized in precooled methanol (*n* = 7, for SOD1^G86R^ mice; *n* = 8, for wild type). Chloroform was added and after centrifugation, organic phase was collected and evaporated. Residues were solubilized with acetonitrile/isopropanol. Chromatography was performed on an Acquity UPLC system using an Acquity BEH C18 column. The chromatographic system was coupled to a LCT Synapt G2S mass spectrometer (Waters Corporation), equipped with an electrospray source operating in positive or negative ion mode with a lockspray interface for accurate mass measurements. Refiner MS 6.0 (Genedata, Basel, Switzerland) was use to normalize metabolomics data. Data with retention times between 3 and 15 min, with a mass range between 300 and 1,000 and peak intensity distinct from zero were retained and normalized to fresh tissue mass. Data were submitted to PARETO transformation before statistical analysis. Statistical analysis for differential lipid metabolite level was performed by using supervised multivariate orthogonal partial least-squares discriminant analysis (OPLS-DA), as implemented in SIMCA-P. A difference was considered significant when the corresponding variable |correlation coefficient| was greated than or egal to 0.7, according to the *p*(corr) coordinate in the S-plot built after the OPLS-DA model. Molecular features with significant changes were associated with theoretically identified metabolites based on their atomic mass (m/z), by using online HMDB 2.5 databases. Together with lipid extraction from samples and liquid chromatography gradient, the brut formula associated with the exact mass-over-ionization-state ratio allowed tentative lipid identification.

### Pathway analysis

Gene enrichment analysis was performed with dysregulated genes (*p*_adj_ < 0.01) by using Gene Ontology/Panther (Ashburner et al., 2000; The Gene Ontology Consortium, 2015). Only the most specific subclass was considered per parent items. Given *p*-value were adjusted using the Bonferroni correction. For lipid metabolites, over-representation analysis was carried out with consensuspathDB (Kamburov et al., 2009, 2011b). All significantly dysregulated and identified lipid metabolites were considered for this analysis. Parameters were set to a minimum overlap of four distinct metabolites and a *p*-value cutoff of 0.001. Pathway over-representation analysis was conducted by using IMPaLA (Kamburov et al., 2011a) with genes and lipid metabolites whose levels were altered in SOD1^G86R^ mice. Pathways represented by at least 3 genes and 3 lipid metabolites and with q-value for enrichment below 0.05 were considered as significantly altered.

## Results

### Gene regulation in the spinal cord of SOD1^G86R^ mice

To gain insight into the nature and extent of metabolic reorientation occurring in ALS, the spinal cord transcriptomes of five 95 days old SOD1^G86R^ and five age- and litter-matched non-transgenic wild type mice were studied by RNA-sequencing. This time point corresponds to the symptomatic disease stage of this animal model. At 95 days of age, all SOD1^G86R^ mice present with motor symptoms and muscle denervation, even if the severity of symptoms may vary (Supplementary Figure 1). Disease onset, defined as a loss of more than 10% of muscle strength is taking place around 90 days of age (Henriques et al., 2017). Disease end-stage, corresponding to full paralysis, occurs later in this mouse line, around 105 days of age.

**Figure 1:**
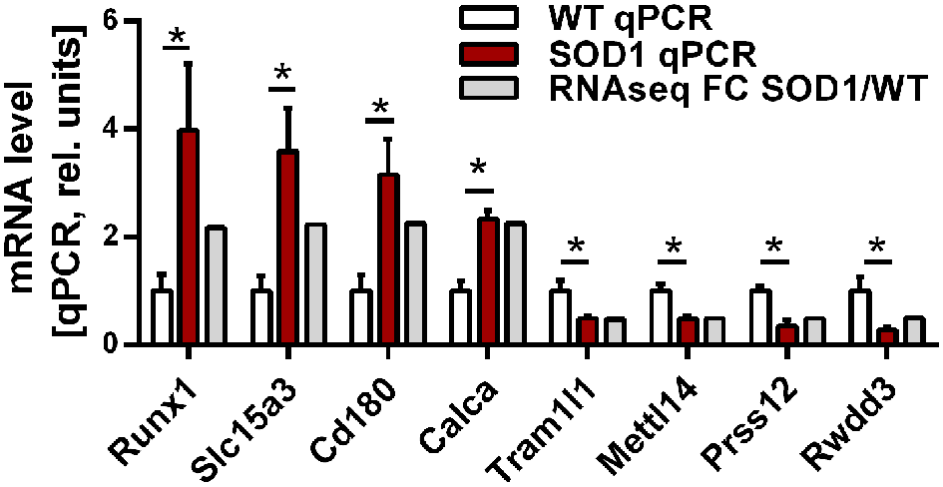
Expression level of selected genes as determined by RNA-sequencing and qPCR. Validation of differential gene expression by qPCR and compared to the fold change determined by RNA sequencing. qPCR values were normalized to the mean of the respective WT group. qPCR data is presented as mean ± standard variation of the mean. FC, fold change; ^*^p < 0.05.

Group comparison identified a total of 751 genes significantly dysregulated (adjusted *p*-value < 0.01), including 660 up-regulations and 91 down-regulations. Table 1 presents all genes found either up- or down-regulated in SOD1^G86R^ mice with a fold change higher than 2 (adjusted *p*-value < 0.01, |log2 fold change| greater than 1). Genes that were the most down-regulated were *Tram1l1* (translocation associated membrane protein 1-like 1), *Rwdd3* (RWD domain containing 3), *Mettl14* (methyltransferase like 14), and *Prss12* (protease, serine 12 neurotrypsin; or motopsin). The highest up-regulation concerned *Sod1* (superoxide dismutase 1, soluble), which is expected due to the presence of the *Sod1*^*G*86*R*^ transgene. *Gm8566* is the second most dysregulated gene. Its altered expression could result from a real difference between SOD1^G86R^ and WT mice, although we cannot completely rule out the possibility of an artifact as *Gm8566* shares 98% homology with the *Sod1* transcript. Aside from the *Sod1* gene and *Gm8566*, highly dysregulated transcripts with known functions were *Cd180* (CD180 antigen), *Calca* (calcitonin/calcitonin-related polypeptide, alpha), *Slc15a3* (solute carrier family 15, member 3), and *Runx1* (runt related transcription factor 1). Differential regulation of gene expression was assessed by quantitative PCR to confirm RNA-sequencing. Selected genes were *Tram1l1, Mettl14, Cd180, Calca, Slc15a3, Runx1, Rwdd3*, and *Prss12*. These genes were selected because they were either the most up-regulated or down-regulated (adjusted *p*-value < 0.01, |log2 fold change| greater than 1) and had known biological functions. Expression levels of the selected transcripts were highly similar when comparing qPCR and RNA-sequencing analysis (Figure 1).

**Table 1:** Transcriptomic dysregulation in the spinal cord of SOD1^G86R^ mice.

**Gene name**	**Description**	**log2 FC**	**Adjusted *p*-value**
Sod1	Superoxide dismutase 1, soluble	4.1	1.5E-254
Gm8566	Predicted pseudogene 8566	3.1	1.2E-101
RP23-182M12.4		1.4	3.6E-15
Cd180	CD180 antigen	1.2	1.3E-11
Calca	Calcitonin/calcitonin-related polypeptide, alpha	1.2	4.6E-21
Slc15a3	Solute carrier family 15, member 3	1.2	1.1E-10
Runx1	Runt related transcription factor 1	1.1	6.7E-11
Slamf9	SLAM family member 9	1.1	4.2E-09
Mmp12	Matrix metallopeptidase 12	1.1	2.6E-09
Bcl3	B cell leukemia/lymphoma 3	1.1	5.1E-09
Slc7a7	Solute carrier family 7, member 7	1.1	1.8E-13
Fcrls	Fc receptor-like S, scavenger receptor	1.1	7.6E-09
Tnfsf8	Tumor necrosis factor (ligand) superfamily, member 8	1.1	1.4E-08
Slfn9	Schlafen 9	1.0	1.9E-08
Glipr1	GLI pathogenesis-related 1 (glioma)	1.0	2.7E-09
Hcar2	Hydroxycarboxylic acid receptor 2	1.0	2.7E-08
Fcgr1	Fc receptor, IgG, high affinity I	1.0	7.6E-09
Ctsh	Cathepsin H	1.0	2.6E-15
Irf8	Interferon regulatory factor 8	1.0	3.0E-09
Arl11	ADP-ribosylation factor-like 11	1.0	5.0E-08
Ctsc	Cathepsin C	1.0	4.1E-11
Apol9a	Apolipoprotein L 9a	1.0	3.3E-08
Cd52	CD52 antigen	1.0	7.9E-08
Egr2	Early growth response 2	1.0	5.6E-08
Klrb1b	Killer cell lectin-like receptor subfamily B member 1B	1.0	5.6E-08
Klhl6	Kelch-like 6	1.0	7.0E-12
Prss12	Protease, serine 12 neurotrypsin (motopsin)	−1.0	1.1E-20
Mettl14	Methyltransferase like 14	−1.1	1.3E-65
Rwdd3	RWD domain containing 3	−1.1	1.8E-23
Tram1l1	Translocation associated membrane protein 1-like 1	−1.1	3.2E-58

*Given is a list of genes showing altered expression level in SOD1^G86R^ mice, with a |log2 fold change (FC)| higher than 1 and with an adjusted p < 0.01*.

**Figure 2:**
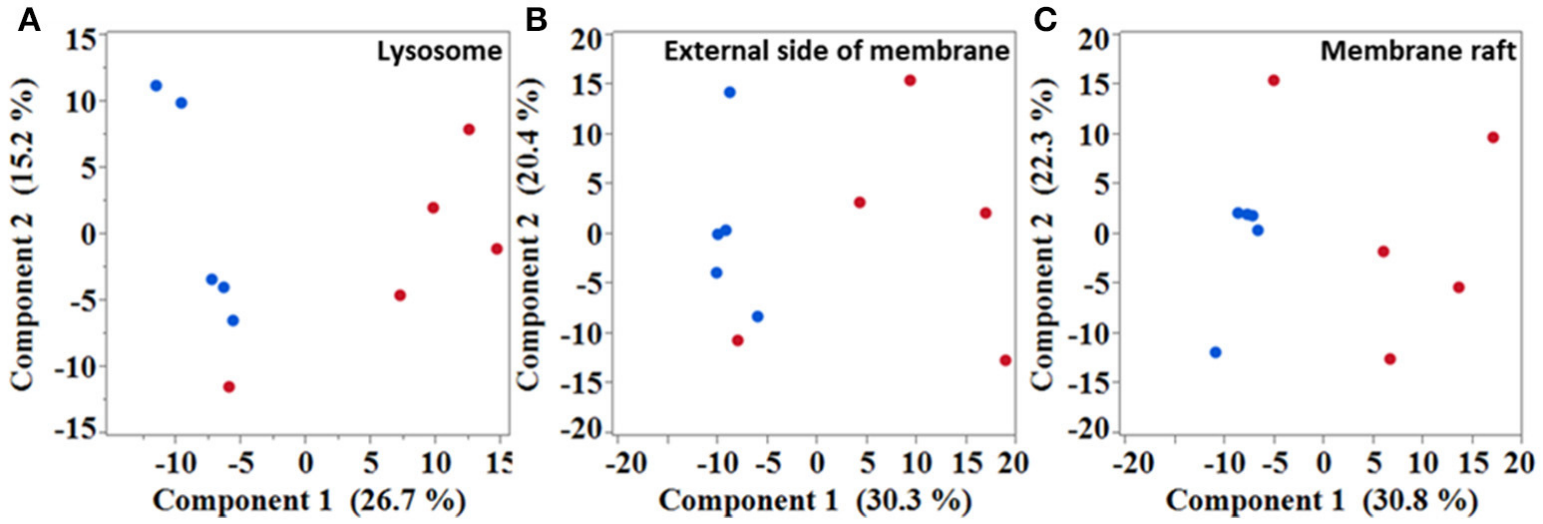
Multivariate analysis showing samples distribution based on different subset of genes. Unsupervised principal component analysis performed with genes isolated from “lysosome” **(A)**, “external side of membrane” **(B)**, and “membrane raft” **(C)** gene ontology terms. Blue dots, wild type; red dots, SOD1^G86R^.

Next, in an attempt to identify biological processes and cellular components affected in the diseased mice, gene ontology (GO) analysis was performed with significantly dysregulated genes. Immune system process was the highest over-represented biological process with a fold enrichment (FE) of 3.9 and an adjusted *p*-value (*p*_adj_) below 4.4.10^−77^. In SOD1^G86R^ mice, 248 genes were associated to this particular biological process, which corresponds to almost of a third of all transcriptomic reorientations. Notably, “positive regulation of signal transduction” (126 genes; FE = 2.87; *p*_adj_ < 2.63.10^−22^), “regulation of locomotion” (96 genes; FE = 3.4; *p*_adj_ < 3.97.10^−21^), “regulation of apoptotic process” (122 genes; FE = 2.5; *p*_adj_ < 3.99.10^−17^), and “response to lipid” (72 genes; FE = 3.7; *p*_adj_ < 4.5.10^−17^) showed strong enrichment in SOD1^G86R^ mice. A putative location of the products of genes showing altered expression was investigated. Over-represented cellular components were mainly associated to membranes or vesicles, such as “extracellular exosome” (170 genes; FE = 2.0; *p*_adj_ < 6.6.10^−16^), “lysosome” (47 genes; FE = 3.0; *p*_adj_ < 6.2.10^−08^), “external side of plasma membrane” (42 genes; FE = 2.89; *p*_adj_ < 2.72.10^−06^), and “membrane raft” (36 genes; FE = 3.18; *p*_adj_ < 3.57.10^−06^). Accordingly, unsupervised principal component analysis (PCA) plots performed with genes from the GO terms “lysosome,” “membrane raft,” and “external side of plasma membrane,” revealed a strong influence of genotype on the distribution of samples (Figures 2A–C). These results suggest that these pathways are strongly impacted in symptomatic SOD1^G86R^ mice.

### Lipidomic changes in the spinal cord of SOD1^G86R^ mice

In order to better characterize metabolomic changes at disease onset, lipid metabolites were studied in seven symptomatic SOD1^G86R^ mice and eight age-matched non-transgenic wild type mice. Analysis of spinal tissue by mass spectrometry identified a total of 7,603 distinct peaks after positive ionization. A total of 293 metabolites was found altered according to the chosen threshold in the SOD1^G86R^ mice compared to wild type controls. A hypothesis-driven identification was attributed to 196 of them accordingly to their atomic mass. All tentatively identified metabolites were down-regulated. An enrichment analysis was performed with the identified and altered metabolites using ConsensuspathDB (Kamburov et al., 2009, 2011b). According to three independent databases, “*sphingolipid metabolism*” (KEGG database, *p*_adj_ < 3.00.10^−05^; and Reactom database, *p*_adj_ < 3.9.10^−05^) as well as “*glycosphingolipid metabolism*” (EHMN database; *p*_adj_ < 2.5.10^−04^) were the metabolic pathways showing the most significant enrichment. Indeed, many metabolites showing altered levels were identified as sphingolipids. Sphingomyelin (d18:0/26:1), ceramide-P (d18:1/26:1), and ceramide (d18:1/22:0) were among the most down-regulated lipid metabolites (Table 2). Notably, several phospholipid metabolites, such as phosphatidylcholine(o-22:1/20:4), presented with significant down-regulation (Table 3). Other subclasses of dysregulated lipids included di- and triglycerides. Saturation and unsaturation of acyl chains directly influence biophysical properties of complex lipids with repercussion on membrane fluidity. According to HMDB identification, down-regulated sphingolipid metabolites mainly quasi-exclusively contained saturated and monounsaturated fatty acids. This suggests that sphingolipids having polyunsaturated acyl chains were not significantly affected in SOD1^G86R^ mice. This difference in acyl chains could reflect enzymatic activities involved in the synthesis, transformation or degradation of fatty acids and lipids.

**Table 2:** Dysregulation of sphingolipid metabolites in the spinal cord of SOD1^G86R^ mice, after positive ionization, and tentative identification.

**m/z**	**RT**	**HMDB**	**Name**	**Fold change**
686.61	12.23	HMDB32794	As 1-1	0.43
624.57	10.41	HMDB11765	Ceramide(d18:0/22:0)	0.57
638.64	11.5	HMDB11767	Ceramide(d18:0/23:0)	0.44
564.54	10.25	HMDB04948	Ceramide(d18:1/18:1)	0.51
594.59	11.59	HMDB04951	Ceramide(d18:1/20:0)	0.52
622.61	10.41	HMDB04952	Ceramide(d18:1/22:0)	0.00
620.62	11.5	HMDB11775	Ceramide(d18:1/22:1)	0.44
636.6	10.82	HMDB00950	Ceramide(d18:1/23:0)	0.54
678.65	10.68	HMDB04955	Ceramide(d18:1/26:0)	0.46
730.61	9.56	HMDB10704	Ceramide-P(d18:1/24:0)	0.54
784.66	10.16	HMDB10707	Ceramide-P(d18:1/26:1)	0.41
784.65	10.92	HMDB10707	Ceramide-P(d18:1/26:1)	0.49
802.65	10.26	HMDB35402	Culinariside	0.51
812.69	11.09	HMDB00140	Glucosylceramide	0.50
812.69	10.83	HMDB00140	Glucosylceramide	0.61
728.61	9.57	HMDB04972	Glucosylceramide(d18:1/18:0)	0.55
784.66	10.93	HMDB04974	Glucosylceramide(d18:1/22:0)	0.53
810.68	10.84	HMDB04975	Glucosylceramide(d18:1/24:1)	0.36
656.57	11.49	HMDB35469	N-(2r-hydroxydocosanoyl)-2s-amino-1,3s,4r-octadecanetriol	0.51
712.6	9.56	HMDB35472	N-(2r-hydroxyhexacosanoyl)-2s-amino-1,3s,4r-octadecanetriol	0.53
536.51	9.6	HMDB35480	N-[(4e,8e)-1,3-dihydroxyoctadeca-4,8-dien-2-yl]hexadecanamide	0.54
650.65	14.07	HMDB00831	N-lignoceroylsphingosine	0.48
650.65	11.08	HMDB00831	N-lignoceroylsphingosine	0.49
538.53	10.27	HMDB00790	N-palmitoylsphingosine	0.48
566.56	10.94	HMDB00829	N-stearoylsphingosine	0.46
786.67	10.92	HMDB11694	Sphingomyelin(d16:1/24:1)	0.52
800.69	11.25	HMDB11696	Sphingomyelin(d17:1/24:1)	0.51
706.58	9.59	HMDB10168	Sphingomyelin(d18:0/16:0)	0.32
781.67	11.25	HMDB13468	Sphingomyelin(d18:0/22:3)	0.46
841.7	10.19	HMDB13461	Sphingomyelin(d18:0/26:1)	0.45
729.61	9.57	HMDB12101	Sphingomyelin(d18:1/18:1)	0.69
785.67	10.92	HMDB12104	Sphingomyelin(d18:1/22:1)	0.53
844.72	12.1	HMDB11698	Sphingomyelin(d18:1/26:0)	0.51
300.29	3.83	HMDB00252	Sphingosine	0.55

*Given are the dysregulated metabolites identified as sphingolipids by the Human Metabolome Database (HMDB), in the spinal cord of symptomatic SOD1^G86R^ mice, after positive identification. m/z, mass to charge ratio; RT, retention time (min)*.

**Table 3:** Dysregulation of phospholipids metabolites in the spinal cord of SOD1^G86R^ mice, after positive ionization, and tentative identification.

**m/z**	**RT**	**HMDB**	**Name**	**Fold change**
762.6	10.9	HMDB07878	PC(14:0/20:0)	0.55
790.7	11.6	HMDB07886	PC(14:0/22:0)	0.56
804.6	10.2	HMDB07953	PC(15:0/22:0)	0.48
832.7	10.2	HMDB07959	PC(15:0/24:0)	0.58
830.7	10.2	HMDB07960	PC(15:0/24:1)	0.56
866.7	10.2	HMDB08191	PC(18:3/24:1)	0.45
738.6	9.6	HMDB08258	PC(18:4/P-16:0)	0.61
764.7	10.9	HMDB08260	PC(18:4/P-18:1)	0.56
902.7	11.3	HMDB08289	PC(20:0/24:0)	0.56
794.7	10.8	HMDB08392	PC(20:3/P-18:1)	0.59
794.6	10.9	HMDB08489	PC(20:4/P-18:0)	0.46
792.7	11.6	HMDB08457	PC(20:4/P-18:1)	0.57
792.7	14.3	HMDB08457	PC(20:4/P-18:1)	0.57
792.7	10.8	HMDB08457	PC(20:4/P-18:1)	0.68
824.7	12.2	HMDB08622	PC(22:2/P-18:1)	0.49
810.6	11.1	HMDB08626	PC(22:4/16:0)	0.63
822.7	11.5	HMDB08653	PC(22:4/P-18:0)	0.47
822.7	11.3	HMDB08653	PC(22:4/P-18:0)	0.56
820.7	11.3	HMDB08654	PC(22:4/P-18:1)	0.52
806.6	10.4	HMDB08725	PC(22:6/16:0)	0.44
806.6	10.2	HMDB08725	PC(22:6/16:0)	0.60
862.7	11.5	HMDB08734	PC(22:6/20:0)	0.49
818.7	12.2	HMDB08755	PC(24:0/14:0)	0.50
856.7	11.3	HMDB08786	PC(24:0/P-18:1)	0.46
866.7	12.3	HMDB08797	PC(24:1/18:3)	0.61
766.7	10.9	HMDB13415	PC(o-16:1/20:4)	0.48
850.7	12.1	HMDB13451	PC(o-22:1/20:4)	0.42
740.6	10.3	HMDB11213	PC(p-16:0/18:3)	0.62
768.6	10.9	HMDB11218	PC(p-16:0/20:3)	0.57
792.6	10.6	HMDB11227	PC(P-16:0/22:5)	0.48
766.6	10.3	HMDB11247	PC(p-18:0/18:4)	0.62
766.6	10.3	HMDB11278	PC(p-18:1/18:3)	0.43
798.7	10.8	HMDB11282	PC(p-18:1/20:1)	0.45
798.7	9.5	HMDB11282	PC(p-18:1/20:1)	0.47
798.7	11.3	HMDB11282	PC(p-18:1/20:1)	0.50
796.7	11.6	HMDB11316	PC(p-18:1/20:2)	0.53
722.5	8.9	ECMDB23488	PE(14:0(3-oh)/19:iso)	0.51
632.5	3.9	HMDB08855	PE(14:1/14:1)	0.45
734.6	10.3	ECMDB23529	PE(19:iso/16:0)	0.54
860.7	11.5	HMDB09501	PE(22:0/22:0)	0.47
782.7	11.3	HMDB09578	PE(22:2/P-18:1)	0.47
780.7	11.3	HMDB09610	PE(22:4/P-18:0)	0.46
816.7	9.7	HMDB11395	PE(p-18:0/24:0)	0.62
750.6	9.6	HMDB11452	PE(p-18:1/20:4)	0.56
765.6	10.2	ECMDB23696	PG(16:0/19:iso)	0.61
765.6	10.3	ECMDB23696	PG(16:0/19:iso)	0.61
823.6	10.4	HMDB10614	PG(18:0/22:6)	0.56
775.6	10.2	HMDB10647	PG(18:2(9z,1)/18:0)	0.58
805.6	10.2	ECMDB23712	PG(19:0cycv8c/19:iso)	0.46
805.6	9.8	ECMDB23712	PG(19:0cycv8c/19:iso)	0.58
737.6	9.6	ECMDB23717	PG(19:iso/14:0)	0.63
807.6	10.4	ECMDB23726	PG(19:iso/19:iso)	0.49
889.6	10.4	HMDB09814	PI(18:0/20:3)	0.58

*Given are the dysregulated metabolites identified as phospholipids by the Human Metabolome Database (HMDB), in the spinal cord of symptomatic SOD1^G86R^ mice, after positive identification. m/z, mass to charge ratio; RT, retention time (min)*.

When considering negative ionization, similar results were obtained. HMDB identification was attributed to 165 lipids metabolites showing significantly altered levels in SOD1^G86R^ mice. A majority of lipid metabolites were down-regulated. Main lipid subclasses were fatty acids, phospholipids (mainly phosphatidylcholine), sphingolipids (e.g., ceramide), and tri-, di-, or mono-acylglycerides (Table 4). Notably, several significantly altered lipid metabolites received an identical identification after both positive and negative ionizations. These lipids were sphingolipids [Cer(d18:1/22:1); Cer-P(d18:1/26:1); Cer(d18:1/18:0)], triglycerides [TG(18:4/15:0/18:4); TG(14:1/20:5/14:1)] or from other lipid subclasses [5-Hydroxy-7-methoxy-2-tritriacontylchromone; N-oleoylethanolamine; Randilongin; Persenone a].

**Table 4:** Dysregulation of lipid metabolites in the spinal cord of SOD1^G86R^ mice, after negative ionization, and tentative identification.

**m/z**	**RT**	**HMDB**	**Name**	**Class**	**Fold change**
311.3	6.5	HMDB02212	Arachidic acid (20:0)	Fatty acid	0.61
309.3	5.3	HMDB02231	Eicosenoic acid (20:1n-9)		0.44
337.3	6.6	HMDB02068	Erucic acid (22:1n-9)		0.51
305.2	3.4	HMDB02925	8,11,14-Eicosatrienoic acid (20:3n-6)		0.34
305.2	3.7	HMDB02925	8,11,14-Eicosatrienoic acid (20:3n-6)		0.45
331.3	3.9	HMDB02226	Adrenic acid (22:4n-6)		0.62
335.3	5.7	HMDB61714	Docosadienoate (22:2n-6)		0.52
335.3	5.6	HMDB61714	Docosadienoate (22:2n-6)		0.53
329.2	3.1	HMDB01976	Docosapentaenoic acid (22n-6)		0.54
333.3	4.7	HMDB02823	Docosatrienoic acid (22:3n-3)		0.42
333.3	4.9	HMDB02823	Docosatrienoic acid (22:3n-3)		0.42
307.3	4.4	HMDB05060	Eicosadienoic acid (20:2n-6)		0.41
307.3	4.3	HMDB05060	Eicosadienoic acid (20:2n-6)		0.49
355.3	3.6	HMDB02007	Tetracosahexaenoic acid (24:6n-12)		0.63
357.3	4.3	HMDB06322	Tetracosapentaenoic acid (24:5n-6)		0.58
359.3	5.1	HMDB06246	Tetracosatetraenoic acid (24:4n-6)		0.57
379.3	6.5	HMDB11545	MG(0:0/20:3n-3/0:0)	Mono, di, triglyceride	0.61
377.3	5.3	HMDB11549	MG(0:0/20:4n-3/0:0)		0.40
405.3	6.6	HMDB11554	MG(0:0/22:4n-6/0:0)		0.51
641.5	11.5	HMDB07119	DG(16:0/22:5n-6/0:0)		0.59
637.5	10.9	HMDB11188	TG(12:0/12:0/12:0)		0.46
845.7	11.9	HMDB42567	TG(14:0/18:3n-3/20:5n-6)		0.48
843.7	11.5	HMDB42807	TG(14:0/18:4n-3/20:5n-3)		0.43
715.6	11.2	HMDB47885	TG(14:1n-5/14:1n-5/14:1n-5)		0.55
791.6	10.9	HMDB47904	TG(14:1n-5/14:1n-5/20:5n-3)		0.50
781.6	10.2	HMDB43186	TG(15:0/14:1n-5/18:4n-3)		0.58
833.7	11.6	HMDB43190	TG(15:0/14:1n-5/22:6n-3)		0.48
831.7	11.6	HMDB43679	TG(15:0/18:4n-3/18:4n-3)		0.43
760.6	9.6	HMDB07878	PC(14:0/20:0)	Phospholipid	0.56
800.6	10.0	HMDB07952	PC(15:0/22:1n-5)		0.54
792.6	9.8	HMDB07956	PC(15:0/22:5n-6)		0.54
828.6	10.2	HMDB07960	PC(15:0/24:1n-9)		0.54
844.7	11.5	HMDB07992	PC(16:0/24:0)		0.54
842.7	10.4	HMDB07993	PC(16:0/24:1n-9)		0.54
744.6	10.9	HMDB07995	PC(16:0/P-18:0)		0.56
772.6	11.6	HMDB08061	PC(18:0/P-18:0)		0.53
884.6	10.3	HMDB08616	PC(22:2n-6/22:6n-3)		0.59
914.7	11.6	HMDB08750	PC(22:6n-3/24:1n-9)		0.54
820.6	10.4	HMDB09212	PE(18:4n-3/24:1n-9)		0.51
758.6	11.9	HMDB09247	PE(20:0/dm18:0)		2.46
719.5	8.5	HMDB10571	PG(16:0/16:1n-9)		0.42
777.6	10.3	HMDB10602	PG(18:0/18:0)		0.59
789.6	9.8	ECMDB23708	PG(18:1n-9/19:iso)		0.53
564.5	9.4	HMDB04950	Ceramide (d18:1n-9/18:0)	Sphingolipid	0.52
566.5	10.1	HMDB11761	Ceramide (d18:1n-9/18:0)		0.63
620.6	10.7	HMDB04952	Ceramide (d18:1n-9/22:0)		0.53
562.5	9.4	HMDB04948	Ceramide (d18:1n-9/18:1n-9)		0.52
562.5	9.5	HMDB04948	Ceramide (d18:1n-9/18:1n-9)		0.52
618.6	10.7	HMDB11775	Ceramide(d18:1n-9/22:1n-13)		0.50
672.5	10.0	HMDB10702	CerP(d18:1n-9/20:0)		0.59
782.6	10.4	HMDB04974	Glucosylceramide (d18:1n-9/22:0)		0.51
886.6	10.9	HMDB11592	Lactosyceramide (d18:1n-9/18:1n-9)		0.46
714.6	10.5	HMDB29216	SM C16:1n-7		0.61

*Given are the dysregulated metabolites identified by the Human Metabolome Database (HMDB), in the spinal cord of symptomatic SOD1^G86R^ mice, after negative identification. m/z, mass to charge ratio; RT, retention time (min)*.

### Sphingolipid metabolism is altered at transcriptomic and lipidomic levels in the spinal cord of SOD1^G86R^ mice

To investigate the relationship between transcriptomic and lipidomic changes in SOD1^G86R^ mice, a joint inter-omics analysis was performed using significantly dysregulated genes and metabolites (Kamburov et al., 2011a). The objective was to identify molecular pathways associated with ALS, with a special regard toward lipid metabolism. Three distinct databases have identified “sphingolipid,” “glycosphingolipid metabolism,” and “sphingolipid signaling” pathways as significantly enriched in the spinal cord of SOD1^G86R^ mice (Table 5, Supplementary Table 2). Overlapping genes are those coding for lysosomal proteins involved in the recycling of glycosphingolipids (e.g., *Hexb, Glb1, Asah1*), for ceramide kinase (*Cerk*), for *Sgpl1*, an enzyme involved in the degradation of sphingosine-1-phosphate. Overlapping lipid metabolites were ceramide, mono-hexosylceramide (glucosylceramide or galactosylceramide), lactosylceramide, and sphingomyelin. Pathways related to other lipids, such as phospholipids or triglycerides, were not detected as significantly enriched.

**Table 5:** Integrated pathway analysis of spinal transcriptomic and lipidomic data of SOD1^G86R^ mice.

**Pathway name**	**Source**	**Overlapping genes**	**Genes**	**Overlapping metabolites**	**Metabolites**	***p*-value**	***q*-value**
Glycosphingolipid metabolism	EHMN	3	Glb1;Asah1;Sgpl1	4	Ceramide (3)	0.00047	0.0125
					Glucosylceramide (4)		
					Sphingosine (1)		
					Sphingomyelin (5)		
Sphingolipid metabolism	KEGG	4	Glb1; Asah1; Cerk; Sgpl1	4	Ceramide (3)	0.0001	0.00062
					Glucosylceramide (4)		
					Sphingosine (1)		
					Sphingomyelin (5)		
Retrograde endocannabinoid signaling	KEGG	6	Gabrq;Gngt2;Adcy7; Plcb2; Gng8;Itpr2	3	Anandamide (1)	0.00057	0.0141
					Phosphatidylethanolamine (4)		
					Phosphatidylcholine (17)		
Sphingolipid signaling pathway	KEGG	11	Ctsd; Asah1; Pik3cg; Sgpl1; Plcb2; Gab2; Rac2; Rac3; S1pr3; Tnfrsf1a; Fcer1g	3	Ceramide (3)	0.0001	0.00297
					Sphingosine (1)		
					Sphingomyelin (5)		

*Given are the metabolic pathways identified as significantly dysregulated in a joint inter-omic analysis performed with IMPaLA. The analysis was performed with the 751 genes and 293 lipid metabolites significantly dysregulated. The table shows the pathways, the source of the database, number and identity of genes and metabolites dysregulated per pathway, and p-value and q-value (false discovery rate) for each pathway*.

**Figure 3:**
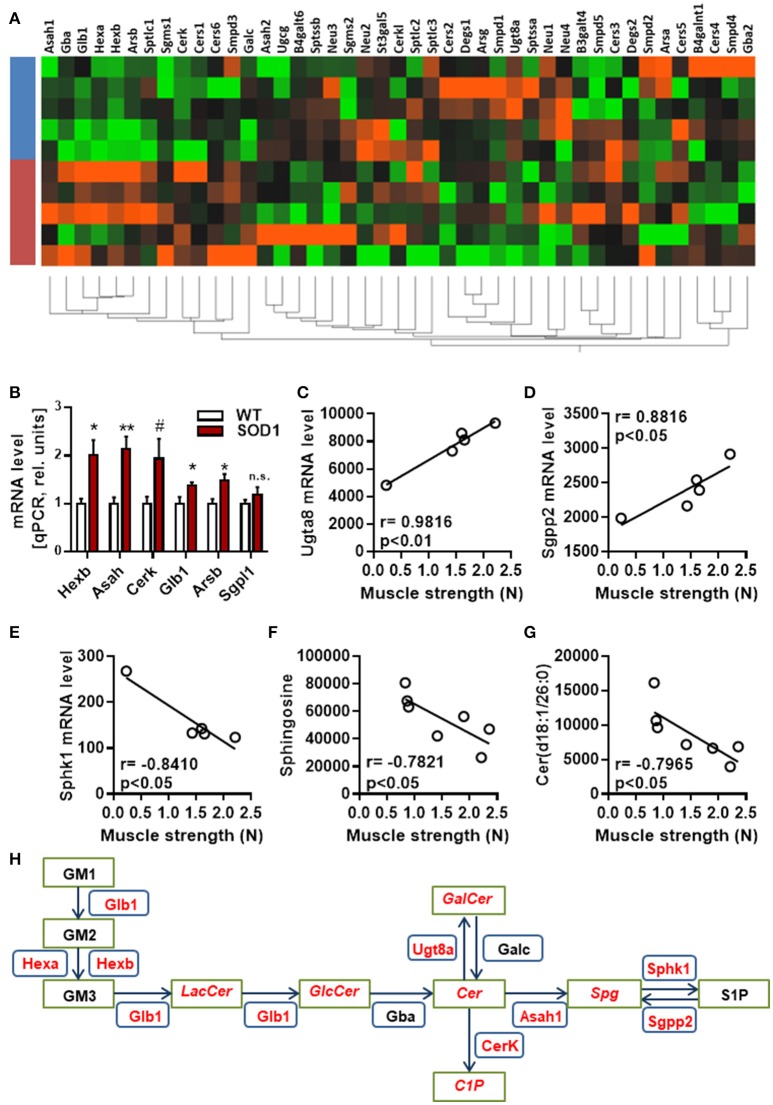
Regulation and interaction of genes related to glycosphingolipid pathway. **(A)** Unsupervised hierarchical clustering, performed with genes from the KEGG pathway “sphingolipid metabolism pathway,” showing distribution of SOD1^G86R^ and wild type samples (blue, wild type; red, SOD1^G86R^). **(B)** Validation by qPCR of differential expression level of genes from the sphingolipid pathway previously found dysregulated by RNAseq. **(C–G)** Pearson correlations between muscle strength and gene expression levels of *Ugt8a* **(C)**, *Sgpp2* **(D)**, *Sphk1* **(E)**, and metabolite levels of sphingosine **(F)** and ceramide(d18:1/26:0) **(G)**. **(H)** Schematic representation of the main dysregulations related to glycosphingolipids, ceramide, and sphingosine. Green boxes represent metabolites and blue boxes refer to genes. In red are given genes and metabolites showing either altered levels or a correlation with disease severity. LacCer, lactosylceramide; GlcCer, glucosylceramide; GalCer, galatosylceramide; Cer, ceramide; Spg, sphingosine; S1P, sphingosine 1-phosphate, C1P, ceramide 1-phosphate. Gene abbreviation follows the *HUGO Gene Nomenclature Committee*-approved gene nomenclature. Data are presented as mean ± standard variation of the mean. n.s, not significant; #*p* < 0.1; ^*^p < 0.05; ^**^p < 0.01.

Next, hierarchical clustering was performed using all genes, dysregulated or not, listed as part of the sphingolipid metabolism by KEGG. Genotype had a strong influence on the distribution of samples on unsupervised hierarchical clustering plot (Figure 3A). A cluster of genes, which are associated with the breakdown of glycosphingolipids, showed a clear differential pattern of expression in SOD1^G86R^ mice compared to wild type controls. Differential expression for genes (i.e., *HexB, Asah1, Cerk, Glb1*, and *Arsb*) involved in the breakdown of glycosphingolipids was confirmed by qPCR (Figure 3B). Muscle weakness is an early event in SOD1^G86R^ mice and ALS patients. SOD1^G86R^ spinal samples were collected at symptomatic disease stage, when motor weakness was established and ranged from mild to severe. In an attempt to identify biological processes influenced by disease severity, correlation between levels of genes or lipid metabolites from the “*sphingolipid metabolism*” and muscle strength was studied. Expression levels of *Ugt8a* (UDP galactosyltransferase 8A), *Sgpp2* (sphingosine-1-phosphate phosphatase 2), and *Sphk1* (sphingosine kinase 1) were significantly correlated or anti-correlated to muscle strength (Figures 3C–E). *Ugt8a* codes for the galactosylceramide synthase. SGPP2 and SPHK1 are two enzymes involved respectively in the synthesis and degradation of sphingosine-1-phosphate.

Interestingly, muscle strength showed a significant correlation between the intensity level of ceramide(d18:1/26:0) and sphingosine, product and/or substrate of *Ugt8a, Sgpp2*, and *Sphk1* (Figures 3F,G). Collectively, our results suggest that the metabolism of sphingolipids, and particularly the glycosphingolipid and the axis “ceramide, sphingosine, sphingosine-1-phosphate” is impaired at transcriptomic and lipidomic levels, and vary depending on disease progression (Figure 3H). Several sphingolipids, such as sphingosine-1-P, ceramide and sphingomyelin, are bioactive molecules able to module cell response to stress, survival, and neuroinflammation. KEGG identified “sphingolipid signaling pathway” as dysregulated, suggesting that altered levels of sphingolipids in ALS could translate into changes in cell signaling.

DGIdb database was used to search for potential pharmaceutical modulators of the “sphingolipid signaling pathway” using genes identified as dysregulated in this pathway (Wagner et al., 2016). A total of 67 compounds were identified, including 10 negative modulators of TNF-a signaling, and 5 modulators of sphingosine-1-phosphate. Notably, 6 of them are currently investigated or were tested at preclinical and/or clinical level in ALS (Pyrimethamine, fingolimod phosphate, pentoxifylline, celastrol, lenalidomide, thalidomide; Supplementary Table 3). These findings indicate that the metabolism of sphingolipid shows dysregulation at gene and metabolites levels, in the spinal cord of symptomatic SOD1^G86R^ mice.

## Discussion

ALS is a fatal condition characterized by degeneration of motor neurons. Several lines of evidence suggest that gene expression and lipid metabolism are differentially regulated in ALS, and could contribute to disease progression. Here, we report a joint analysis of lipidomic and transcriptomic changes in the spinal cord of symptomatic SOD1^G86R^ mice.

### Transcriptomic changes in spinal cord from SOD1^G86R^ mice

For the first time, we report RNAseq analysis of spinal tissue from SOD1^G86R^ mice. This specific mouse line presents with the advantage to overexpress a mutated SOD1 protein without dismutase activity. Based on our transcriptomic analysis, spinal cord of SOD1^G86R^ mice was characterized by the dysregulation of immune system, cell death and regulation of membrane and vesicles. “Immune system” was the biological pathway showing the greatest enrichment in SOD1^G86R^ mice. Numerous studies pin point neuroinflammation in ALS. Activation of glial cells has been linked to degeneration of motor neurons (Yamanaka et al., 2008; Endo et al., 2015; Cooper-knock et al., 2017) or to defects in the integrity of peripheral motor axons (Nardo et al., 2016). Infiltration of peripheral monocytes and lymphocytes into CNS was also demonstrated in ALS (Hooten et al., 2015). Among dysregulated genes identified in our RNAseq analysis, several genes are directly connected to a pro-inflammation state. For instance, *Cd180*, one of the most up-regulated gene in the spinal cord of SOD1^G86R^ mice, is a pro-inflammatory gene participating in the activation of TLR4 receptors (Bastiaansen et al., 2014). Our results also show that *Gfap* (log2FC: 0.54, adjusted *p*-value < 0.01) and *Aif-1/Iba1* (FC: 0.77, adjusted *p*-value < 0.01), markers for astrocyte and microglial activations, were both up-regulated in our study. Interestingly, we noted an up-regulation for *Htr2b* (log2FC: 0.34; adjusted *p*-value < 0.05), gene coding for 5-HT2b receptor, in SOD1^G86R^ mice. A recent report connected serotonin signaling in microglial cells to neuroinflammatory process and neurodegeneration in ALS patients and animal models (El Oussini et al., 2016). *Csf3r* (log2FC: 0.96; adjusted *p*-value < 0.01) and *Csf2rb* (log2FC: 0.96; adjusted *p*-value < 0.01), coding for the receptors of granulocyte (G-CSF) and granulocyte-macrophage stimulating factor (GM-CSF), were significantly up-regulated the SOD1^G86R^ group. The hematopoietic factor G-CSF, ligand of Csf3r, is neuroprotective in SOD1^G93A^ mice (Henriques et al., 2011) and is able to re-adjust gene expression in lumbar motor neurons (Henriques et al., 2015c).

Our analysis also identified genes whose regulation could promote neurodegeneration or impair neuronal function in SOD1^G86R^ mice. *Calca*, encoding for calcitonin gene-related peptide, is up-regulated in our RNAseq analysis. This gene is tightly associated to neurodegeneration and to motor neuron function (Enjin et al., 2010). In spinal bulbar muscular atrophy, its overexpression triggered cell damage while its inhibition reduced, or even abolished, neurodegeneration in an animal model of this disease (Minamiyama et al., 2012). High expression level of *Calca* has been also associated to higher motor neuron loss in SOD1^G93A^ mice (Ringer et al., 2012). *Prss12*, or motopsin, is a neuronal protease which positively regulates axonal plasticity (Mitsui et al., 2013). Prss12 expression is known to be down-regulated during acute stress (Numajiri et al., 2006) and in laser-captured SOD1^G93A^ motoneurons (Henriques et al., 2015c). Down-regulation of *Prss12* in SOD1^G86R^ mice could therefore relate to axonal dysfunction. *Rwdd3* is a gene coding for RSUME, a protein taking part in HIF-1 alpha signaling (Carbia-nagashima et al., 2007) and known to be activated during cell stress. RSUME modulates the sumoylation of the glucocorticoid receptor (Druker et al., 2013), connects to immune system, energy metabolism and axonal plasticity. RSUME down-regulation in spinal tissues of SOD1^G86R^ mice could negative impact motor units through these biological processes. *Runx1* is a transcription factor stimulating neuronal differentiation and axonal plasticity and reduces BMP signaling and expression of *Calca* (Yoshikawa et al., 2015, 2016; Halevy et al., 2016). *Runx1* is a positive regulator of the expression of genes related to sphingolipid metabolism (Kilbey et al., 2010). Thus, the up-regulation of *Runx1* in the spinal tissue of SOD1^G86R^ mice could represent a compensatory neuroprotective mechanism.

Main altered cellular component were “extracellular exosome” and “lysosome.” Exosomes are vesicles releasing proteins, lipids and RNA in the extracellular environment. Involvement of exosome in ALS has been recently a subject of extensive studies. Exosomes participate in the propagation of misfolded SOD1 and TDP-43 proteins (Nonaka et al., 2013; Grad et al., 2014), in aberrant phenotype of immune cells (Pinto et al., 2017) and could impair synaptic plasticity in neurodegenerative diseases (Wang et al., 2017). In fly neuromuscular junctions, presynaptic exosomes transfer synaptotagmin 4 to post-synaptic cells, thereby enabling retrograde signaling in activity-dependant synaptic growth (Korkut et al., 2013). Physiological adaptations to exercise have been proposed to be mediated, at least partially, by muscle-derived exosomes (Safdar et al., 2016). Lysosomes are intracellular organelles with important function in recycling of macromolecules, including complex lipids. Lysosomes interact with autophagosomes to promote autophagy. Defect in lysosomal function results in pathological accumulation of biomolecules, such as sphingolipids, and cause lysosomal storage diseases, such as Gaucher's disease. In ALS, activity of lysosomal enzymes is increased in post-mortem spinal tissues of ALS patients (Dodge et al., 2015) and mutations on genes related to autophagy are causing genetic forms of ALS (Lee et al., 2015).

### Lipidomic changes in spinal cord from SOD1^G86R^ mice

At disease onset, SOD1^G86R^ mice presented with clear rearrangement of lipid metabolites, such as phospholipids and sphingolipids. We and other previously reported that nervous tissue of SOD1 ^G86R^ and SOD1^G93A^ mice had altered levels for lipid metabolites, at presymptomatic, or late-symptomatic disease stages (Cutler et al., 2002; Dodge et al., 2015; Henriques et al., 2015b; Patin et al., 2016). Due to technical restrictions, most of these studies focussed on a limited number of lipid metabolites. In the present study, we have detected more than 7,500 metabolites which covered main lipid subclasses (fatty acids, triglycerides, phospholipids, sphingolipids, and sterols). Shortage in energetic lipids (e.g., triglyceride) is compatible with increased energy expenditure which negatively impact prognosis in ALS patients (Jésus et al., 2018). We have previously reported that triglycerides were massively depleted in spinal cord, muscle, and plasma samples, showing the scale of the metabolic dysfunction in this animal model of ALS (Henriques et al., 2015b). Our data also shows that sphingolipids and phospholipids are under-represented. These lipid classes are key components of the cell membrane.

Changes in proportion of phospholipids could result from a defect in synthesis or higher degradation by phospholipases. Interestingly, we noted an up-regulation of lysosomal phospholipase A2 (Pla2g15; log2FC: 2.56; adjusted *p*-value < 0.01), phospholipase C, beta 2 (Plcb2; log2FC: 1.75; adjusted *p*-value < 0.001), phospholipase C, epsilon 1 (Plce1; log2FC: 1.67; adjusted *p*-value < 0.001), phospholipase C, gamma 2 (log2FC: 2.38; adjusted *p*-value < 0.01), and phospholipase D4 (log2FC: 1.04; adjusted *p*-value < 0.001). It is therefore plausible that the loss in phospholipids could be due, in part, to higher expression, and potentially higher activity, of phospholipases. We noted that sphingolipids with saturated and mono-unsaturated acyl chain were prone to be dysregulated. The nature of acyl chains in complex lipids may reflect dietary habits as well as the state of peripheral energy metabolism. High content in saturated fatty acids is often associated with increased basal metabolism. Lipidomic analysis in yeast revealed a regulatory role for glucose-sensing pathways in acyl chains in phospholipids (da Silveira Dos Santos et al., 2014). Altered levels of saturated fatty acids were described in the cell fraction of blood of ALS patients and higher content in monounsaturated fatty acids was associated with higher survival (Henriques et al., 2015a). Unsaturation impacts membrane fluidity and low proportion in monounsaturated acyl chains leads to higher stiffness. Disturbance in membrane fluidity is known to impair cellular function such as cell signaling.

### Sphingolipids in spinal cord from SOD1^G86R^ mice

Here, we describe, for the first time, common dysregulations in the spinal cord of an animal model of ALS, at gene and lipid levels. Our analysis identified sphingolipid metabolism as the most dysregulated biological pathway. Sphingolipids are complex lipids derived from sphingosine.

These lipids act as structural lipids, signaling molecules (e.g., ceramide, ceramide-phosphate) or as ligands for cell membrane receptors (e.g., spingosine-1-P, lysophosphatidic acid). The causes behind the modifications of sphingolipid metabolism could be multiple. Sphingolipids are involved in key pathways for ALS, such as autophagy and protein clearance, cell survival, energy metabolism, and neuroinflammation. In yeast, the ceramide synthase genes are termed “*lag*-genes,” for “longevity-associated genes” regulating nutrient sensing, autophagy and lifespan. However, excess in ceramide in rodents promotes accumulation of lipids, triggers endoplasmic reticulum, and lipotoxic stress (Martínez-Sánchez et al., 2017). In multiple sclerosis, increased level of ceramides is suspected to contribute to mitochondrial dysfunctions and axonal damage (Vidaurre et al., 2014). Loss of ceramide in the CNS of SOD1^G86R^ mice could therefore directly impact autophagy process, cell bioenergetics and neuronal integrity. Sphingosine-1-phosphate exerts trophic effects on neuron (Miguez et al., 2015; Geffin et al., 2017) but also on muscle during acute stress such as denervation (Danieli-Betto et al., 2005; Zanin et al., 2008).

Similarly, ceramide-phosphate promotes cell survival through the PI3-K/PKB pathway (Gómez-Muñoz et al., 2005; Gómez-Muñoz, 2006). Interestingly, the upregulation of *Cerk* and *Sphk1*, two genes involved in the synthesis of ceramide-phosphate and sphingosine-1-phosphate could be a protective, or compensatory, mechanism for counteracting neurodegeneration. Our results reinforce the critical role of complex lipids, which are dysregulated, in neuronal stress.

At disease onset, SOD1^G86R^ mice present with marked down-regulation of monohexosylceramide (glucosylceramide or galactosylceramide), and lactosylceramide. Similarly to phospholipids, lower levels of these lipids could be due to lower synthesis or higher degradation/conversion. *Ugt8a* codes for the galactosylceramide synthase and was found down-regulated in SOD1^G86R^ mice. Interestingly, its expression level correlated to disease severity in SOD1^G86R^ mice. It suggests a reduced synthesis of galactosylceramide in SOD1^G86R^ mice, and depends on disease severity. Galactosylceramide and glucosylceramide are precursors of sulfatides and gangliosides, which are particularly abundant in the CNS. Gangliosides in particular are enriched in lipid rafts and contribute to signal transduction. For instance, ganglioside GM1a interacts with TrkA and potentiates BDNF signaling and neuroprotection. Defect in GM1a-TrkA signaling could lead to serious neuronal phenotypes as this complex has been proposed as a binding site for toxins from clostridium perfringens (Oda et al., 2012). Dysregulated glycosphingolipid metabolism could therefore participate in the progression of ALS symptoms in SOD1^G86R^ mice.

### Clinical perspectives for ALS

One issue with data obtained with the SOD1 mice is the translatability to clinic. Many clinical trials, based on preclinical data obtained with the SOD1 models, have failed in ALS. This has raised concerns regarding the reliability of the SOD1 mice as models for ALS. In our opinion, the SOD1^G86R^ line remains a good experimental tool for studying ALS. Indeed, these mice present with the main motor and metabolic symptoms found in ALS patients, although only 2% of patients harbor mutations on the *Sod1* gene. Regarding lipid metabolism, both ALS patients and SOD1^G86R^ mice present higher incidence of hypermetabolism (Dupuis et al., 2004; Funalot et al., 2009) and with similar increases in glycosphingolipids in the CNS, such as GM1a (Dodge et al., 2015; Henriques et al., 2017).

Riluzole and edaravone are two drugs approved for the treatment of ALS in the USA. They both have beneficial effects on motor functions and survival in the SOD1^G93A^ mice (Gurney et al., 1996; Waibel et al., 2004; Ito et al., 2008; Del Signore et al., 2009; Shin et al., 2012), although riluzole's effects in SOD1 mice are questioned as they were not confirmed in other studies (Scott et al., 2009; Li et al., 2013; McAllum et al., 2013). Discrepancies between studies could originate from the experimental protocols (e.g., dose, route of administration) or from low rigorousness in study design (e.g., sample size, blinding of experimenters) (Scott et al., 2009; Ludolph et al., 2010). Nutritional interventions, which have shown promising results in ALS patients (Dorst et al., 2013; Wills et al., 2014), extend significantly the survival of SOD1^G86R^ mice (Dupuis et al., 2004). Therefore, we believe that the SOD1 models, and particularly the SOD1^G86R^ model, remain predictive tools for studying pathological processes of ALS.

Our study highlights dysregulations of sphingolipid metabolism in SOD1^G86R^ mice at symptomatic disease stage. As bioactive molecules, sphingolipids interplay with many cell pathways that could be targeted with drug candidates. Pharmacological modulators targeting the metabolism of sphingolipids exist and have been proposed for the treatment of human diseases (Canals et al., 2011). *In silico* analysis has identified fingolimod and pyrimethamine as tentative modulators for ALS. These two drugs modulate sphingosine-1-phosphate signaling and have been already tested in ALS. Fingolimod improved motor functions and survival of SOD1^G93A^ mice and has been given to ALS patients in a clinical phase 2a safety study. Pyrimethamine has been shown to successfully lower mutant SOD1 proteins in the cerebrospinal fluid of ALS patients carrying SOD1 mutations. Other potential drug candidates have been tentatively identified and most of them have not been investigated in ALS. The facts that our data-driven analysis identified pharmacological compounds already tested in ALS reinforce the relevance of our results.

To conclude, spinal cord of SOD1^G86R^ mice present with profound dysregulations at transcriptomic and metabolomic levels that point to sphingolipids. Our results complement existing data on dysregulation of complex lipids in ALS and could open new therapeutic strategies linked to sphingolipids.

## Author contributions

AH, VC, CK, MS, J-PL, and BW: Conceived and designed the experiments; AH, VC, AB, AM, and CK: Performed the experiments; AH, VC, AB, AM, CK, BW, CB-N, MS, and J-PL: Analysis and interpretation of the data; VC, CB-N, BW, CK, MS, and J-PL: Contributed reagents, materials, and analysis tools; AH, VC, AB, AM, CK, BW, CB-N, MS, and J-PL: Wrote, discussed and approved the final version of the manuscript.

### Conflict of interest statement

AH and MS are employees of Spedding Research Solutions SAS. VC, BW, and CB-N are employees of Les Laboratoires Servier. The other authors declare that the research was conducted in the absence of any commercial or financial relationships that could be construed as a potential conflict of interest. The funders had no role in study design, data collection and analysis, decision to publish or preparation of the manuscript.
