# Supplementary material for: Sphingolipid Metabolism Is Dysregulated at Transcriptomic and Metabolic Levels in the Spinal Cord of an Animal Model of Amyotrophic Lateral Sclerosis
Source: Front Mol Neurosci. 2018 Jan 4;10:433. doi: 10.3389/fnmol.2017.00433 (PMC5758557; doi:10.3389/fnmol.2017.00433)
Supplement: Supplementary file 4 [file Presentation1.pptx]

## Slide 1
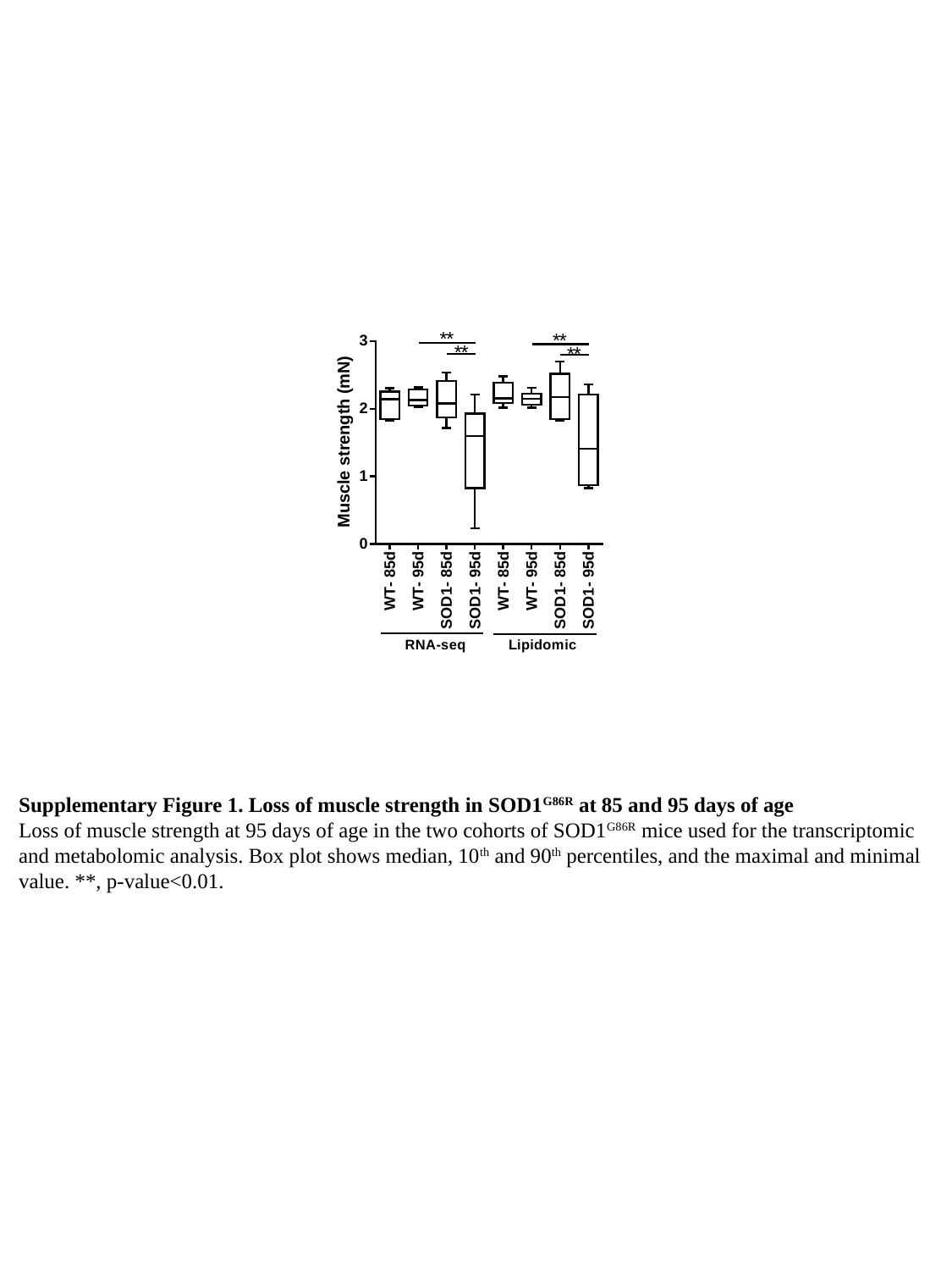

Supplementary Figure 1. Loss of muscle strength in SOD1G86R at 85 and 95 days of age
Loss of muscle strength at 95 days of age in the two cohorts of SOD1G86R mice used for the transcriptomic and metabolomic analysis. Box plot shows median, 10th and 90th percentiles, and the maximal and minimal value. **, p-value<0.01.
